# Factors Related to Gender Violence and Sex Education in Adolescents: A Cross-Sectional Study

**DOI:** 10.3390/ijerph18115836

**Published:** 2021-05-29

**Authors:** Cristina Guerra-Marmolejo, Eloísa Fernández-Fernández, María González-Cano-Caballero, Marina García-Gámez, Francisco J. del Río, Eloisa Fernández-Ordóñez

**Affiliations:** 1 Department of Nursing, Faculty of Health Sciences, University of Málaga, 29071 Málaga, Spain; cguerra@uma.es (C.G.-M.); magagadue@uma.es (M.G.-G.); eloisafernandez@uma.es (E.F.-O.); 2 Agencia Sanitaria Costa del Sol, Costa del Sol Hospital, 29603 Marbella, Spain; eloisa.fernandez.fernandez@juntadeandalucia.es; 3 Department of Nursing, School of Nursing, Physiotherapy and Podiatry, University of Seville, 41009 Seville, Spain; 4 Department of Psychology, Institute of Biomedical Research and Innovation of Cádiz (INIBICA), University of Cádiz, Puerto Real, 11519 Cádiz, Spain; franciscojavier.delrio@uca.es

**Keywords:** sexual health, nursing, school medical service, ambivalent sexism, sexual double standards, romantic love myths, adolescent, gender violence, risk factors

## Abstract

Background: For school medical services and the staff responsible for sex education for adolescents, it is important to understand the factors that may influence gender violence. The aim of this study is to determine whether the presence of sexist attitudes, double standards and/or romantic myths contributes to the risk of gender violence. Methods: This cross-sectional study was carried out at five secondary schools in the province of Malaga (Spain). In total, 879 adolescents aged 12–18 years were included, studying years 1–4 of compulsory secondary education. Their attitudes were measured on the following scales: Ambivalent Sexism Inventory (ASI), Double Standard Scale (DSS) and Romantic Love Myths Scale (EMA). Results: Significant differences were observed among the age/year groups for the mean scores obtained on each of the above scales (DSS, *p* < 0.01; EMA, *p* < 0.01; ASI, *p* < 0.01). By gender, the boys recorded higher scores for ASI and lower ones for DSS (*p* < 0.01). The Spearman’s rho value revealed significant relationships between the presence of sexual double standards and that of romantic myths and ambivalent attitudes (*p* < 0.01). Conclusions: Adolescents commonly express romantic love myths, sexist attitudes and sexual double standards. These three factors, which are significantly correlated, influence the presence of violence in dating relationships.

## 1. Introduction

For health educators and school medical services, it is important to understand the factors that may influence sexual health and gender violence in adolescence. Intimate partner conflicts can become violent, and relationships between adolescents may also present this behavior [1]. Many national and international organizations have published data showing that violence against women is a major social, health, and political issue, affecting all areas of society. The World Health Organization (WHO) has defined it as a global public health problem and the 1979 UN Convention on the Elimination of All Forms of Discrimination against Women describes this violence as an attack on human rights [2].

Aggression in society and intimate partner violence have become increasingly common, as overexposure and banalization have led to violent behaviors seeming less aberrant. This trend is provoking great concern among health education policymakers and practitioners [3]. According to research studies and the WHO, 30% of adolescents have suffered violence within a romantic relationship [4], and slightly fewer in Spain [5]. Dating violence first appears in adolescence and causes a severe impact, both immediately and in the long term, on victims’ quality of life. Moreover, it increases the risk of gender violence in subsequent relationships [6,7].

The term teen dating violence refers to any form of violent behaviors (physical, psychological, or sexual), including harassment and threats, by adolescents towards their partners [6]. Generally mediated by age, this gender violence tends to progress gradually, and its continuing existence or development is subject to factors such as the justification of violence as a means of conflict resolution. In this respect, as in all forms of violence, in gender violence against women, associated (inter- or intra-) personal and situational variables can play a precipitating, facilitating, mediating/modulating or inhibiting/protective role. Among factors that may precipitate gender violence against women are the concurrent consumption of alcohol and other drugs, jealousy and controlling behaviors, risky sexual practices, and unwanted pregnancy [8,9]. It is also more likely following exposure to models of violent behaviors [7]. 

Gender violence is strongly related to sexist attitudes, romantic love myths, and sexual double standards, which are often used to justify and explain attitudes of control or jealousy (rather than condemning them), since they are held to be associated with masculine attributes based on the predominance of men over women or with manifestations of bonding [10,11]. Additionally, gender differences are perpetuated by ambitions of social dominance among humans. Despite real advances in the construction of gender equality, patterns of sexist socialization and double standards persist and are reproduced in successive generations, partly due to inadequate sexual health education, within the family and at school [12,13].

Today the term ambivalent sexism, coined by Glick and Fiske [14], is used, referring to the coexistence of positive attitudes towards women and sexist antipathy. This situation presents two differentiated components: hostile sexism (the view that women are inferior and hence subordinate) and benevolent sexism (nevertheless, women may play certain roles). Healthcare personnel should realize that this ideology is not neutral, but seeks to maintain the status quo and perceived female subordination [15]. Societies with the highest rates of sexism also have the highest rates of violence against women [16]. In consequence, hostile sexism is observed more frequently among males, young and old, than females [14,16,17]. Studies have highlighted the role played by ambivalent sexism in the perception and acceptance of discrimination against women and in the physical and sexual violence exerted within an intimate relationship, giving rise to major problems of public health [16,18]. Intercultural studies have shown that the two components of ambivalent sexism represent complementary ideologies by which their proponents seek to maintain traditional gender roles and power relations [19]. The outcome is greater gender inequality, the continuation of the status quo in society, and the persistence of a rigid gender hierarchy, characterized by double standards [16].

Likewise, another important factor in the sexual education of adolescents is the impact of romantic love myths or the set of socially shared beliefs about the supposed true nature of love. These beliefs can form solid patriarchal foundations and become firmly and unquestioningly established in the mindset. Indeed, they may even be present, albeit indirectly, in the health education received [20]. The assimilation of such ideas is the perfect breeding ground for the development of tolerance towards abuse, thus ensuring that historical levels of gender violence continue to be breached year after year [15,21] and blurring the lines between what adolescents consider to be abuse and that normal behavior [22].

With these considerations in mind, those responsible for sex education and the promotion of healthy sexual behaviors should be aware of the influence of these factors on gender violence among adolescents, and on differences in perceptions according to gender. Our main study aim is to analyze the presence of romantic love myths among adolescents, their expressions of sexist attitudes, and sexual double standards towards women. The specific objectives are to describe the differences in the cited variables based on the sociodemographic characteristics of the sample and to analyze the interrelationship between these variables. The scientific hypothesis of the present study is that the three variables that influence gender violence (ambivalent sexism, sexual double standards, and romantic love myths) are correlated with each other.

## 2. Materials and Methods

### 2.1. Study Design

This cross-sectional study [23], part of a larger study, was carried out in secondary schools in the province of Malaga (Spain).

### 2.2. Population

The reference population was composed of adolescents living in the province of Malaga. The sample size required (857 individuals) was calculated assuming a confidence level of 99%, *p* = q = 0.5, a precision of 5% and a replacement rate of 30%. The inclusion criteria applied were that the students should have attended the class in which the questionnaires were distributed, that their parent or guardian had authorized their participation and that their participation in the study was completely voluntary. Any students (*n* = 6) who had difficulty in reading and/or understanding the questionnaires, due to language reasons or due to impaired cognitive abilities, were excluded. The study had a response rate of 95% of the participants.

### 2.3. Measurement Instruments

An ad hoc questionnaire was used to collect the sample’s sociodemographic variables. The data collected were age, gender, school year, place of residence, sexual orientation, having a partner or not, and age they were when they had their first sexual relationship.

The following validated scales were used:

Ambivalent Sexism Inventory (ASI), designed by Glick and Fiske [14] and adapted to Spanish by Expósito, Moya and Glick [24], validated in adolescents by Ibabe, Arnoso and Elgorriaga [25]. The ASI evaluates ambivalent sexist attitudes (hostile and/or benevolent) towards women. It consists of 22 Likert-type response items, scored from 0 (Totally disagree) to 5 (Totally agree). The reliability of the Spanish validation, in two studies, was 0.90 and 0.88, respectively (ASIb 0.86 and ASIh 0.89). The theoretical mean (2.5) is used as a cut-off point to differentiate between low and high ambivalent sexism (benevolent and hostile). ASI is made up of two subscales, benevolent sexism (items 1, 3, 6, 8, 9, 12, 13, 17, 19, 20, 22) and hostile sexism (items 2, 4, 5, 7, 10, 11, 14, 15, 16, 18, 21). ASIh represents a single dimension and ASIb is made up of three different aspects: protective paternalism, complementary gender differentiation, and heterosexual intimacy. The reliability in the present investigation is 0.88 (ASIb α = 0.79 and ASIh α = 0.83).

Double Standard Scale (DSS), designed by Caron et al. [26], adapted into Spanish by Sierra et al. [27] and validated in Basque adolescents by Ubillos et al. [28]. This unifactorial scale evaluates assessments of sexual behaviors according to the gender of the person making the assessment. It consists of ten Likert-type response items, scored from 1 (Totally agree) to 5 (Totally disagree). The reliability of the Spanish validation is 0.76 in men and 0.70 in women. The reliability in the present investigation is 0.83. 

Romantic Love Myths Scale (EMA, Spanish initials), designed by Bosch et al. [29], validated in adolescent by Rodríguez-Castro et al. [30]. This scale evaluates the presence and strength of myths about love. It consists of eight Likert-type response items, scored from 1 (Completely disagree) to 5 (Completely agree). The reliability of the original sample was 0.506. EMA is made up of two factors: factor 1, α = 0.525, idealization of love (items 1, 2, 3, 4, 5, 8), and factor 2, α = 0.645, link between love and abuse (items 6, 7). The reliability in the present investigation is 0.65 (idealization of love α = 0.52, and link between love and abuse α = 0.72).

### 2.4. Sampling and Recruitment

Participants in this study were recruited non-probabilistically by quota sampling, conducted in secondary schools in the province of Malaga. To ensure an appropriate sample size, five schools were selected, differentiating between urban and rural, since this might be a confounding variable. If any school declined to participate, another from the same area was randomly selected. Once they accepted the Secondary Schools, they were passed to all the students of all the classes that belonged to ESO. The data collection process was strictly confidential and participation in the study was voluntary in all cases.

### 2.5. Procedure

Authorization was requested from the School Councils of the Secondary School in which the questionnaires were to be approved. Written authorization from parents was requested, as well as oral authorization from adolescents. Classrooms were attended during class time, and each student was given a booklet with the paper questionnaires. They took approximately 15 min to complete the questionnaires.

The study was carried out in accordance with the guidelines of the Declaration of Helsinki and was approved by the Ethics Committee of the University of Malaga (9-2021-H). The protection and confidentiality, contained in Organic Law 3/2018, of December 5, on the Protection of Personal Data and guarantee of digital rights, was guaranteed. In addition, authorization was requested from the School Councils of the secondary schools, as well as the signed consent of the parents and students.

### 2.6. Statistical Analysis

Descriptive analyses were performed to obtain the corresponding mean values and standard deviations or percentages, according to the nature of the variables. The Kolmogorov–Smirnov test was used to analyze the normality of the sample data distribution, and relationships between the variables were determined by the Spearman bivariate correlation test. The Kruskal–Wallis and Mann–Whitney U tests were used to confirm the significance or otherwise of the differences in the mean scores obtained from the questionnaires. We conducted data analysis on SPSS 25.0 (International Business Machines Corporation, Armonk, NY, USA).

## 3. Results

We carried out this research in secondary schools. In total, 53 students (6.03%) were studying in their first year of Compulsory Secondary Education (Educación Secundaria Obligatoria (ESO), Spanish initials), 136 (15.47%) were in their second year, 347 (39.48%) were in the third year and 343 (39.02%) were in their fourth year. The descriptive analysis of the variables is reflected in Table 1 and Table 2.

The sample was sub-divided by sociodemographic characteristics in order to analyze the attitudes and beliefs professed. Table 3 shows the questionnaire scores obtained, according to school year, gender, the existence/absence of a relationship and residence place. Among other findings, the students in the first year of ESO scored highly for the EMA scales and ASI scales, but not for DSS. By gender, the boys scored more highly on the sexism scales and EMA scales, while the girls scored more highly on the DSS. 

The Kolmogorov–Smirnov test showed that none of the questionnaire results met the normality criterion (DSS: *p* = 0.007; EMA: *p* = 0.007; EMAi: *p* < 0.001; EMAa: *p* < 0.001; ASI: *p* = 0.021; ASIb: *p* = 0.032; ASIh: *p* = 0.014). 

The non-parametric Kruskal–Wallis test revealed significant differences between the mean scores obtained according to the school year considered, for each of the scales analyzed: DSS, H = 31.94, df = 3 (*p* < 0.01); EMA, H = 45.70, df = 3 (*p* < 0.01); EMAi, H = 22.03, df = 3 (*p* < 0.01); EMAv, H = 92.98, df = 3 (*p* < 0.01); ASI, H = 57.14, df = 3 (*p* < 0.01); ASIb, H = 54.50, df = 3 (*p* < 0.01); ASIh, H = 35.53, df = 3 (*p* < 0.01). The Kruskal–Wallis test adjusted with the Bonferroni correction was carried out, to see the differences between matched school grades (Table 4). For the interpretation of the effect size measures, we took into account the Cohen’s d ranges: <0.2 “no effect”, 0.2–0.5 “small effect”, 0.5–0.8 “medium effect”, and >0.8 “great effect”.

It can be observed that there are significant differences between 1st year and all the other school years in relation to sexual double standards, which indicates that they have a greater acceptance of sexual double standards. In turn, there are significant differences between 4th year with respect to 1st year and 2nd year in romantic myths, ambivalent sexism, and sexual double standard, so that the younger adolescents have greater adherence in all factors. 

Finally, the Mann–Whitney test was performed to verify the differences according to gender, relationship status and location. According to gender, there were significant differences (*p* < 0.01) in DSS (Z = −4.816), EMAa (Z = −3.819), ASI (Z = −3.135), ASIb (Z = −3.135), and ASIh (Z = −9.153). Based on relationship status, there were significant differences only for EMA (Z = −2.202, *p* < 0.05) and EMAi (Z = −2.857, *p* < 0.01). Relative to location, there were significant differences (*p* < 0.01) in DSS (Z = −4.829), EMA (−6.507), EMAi (Z = −4.384), EMAa (Z =−8.377), ASI (Z = −4.090), ASIb (Z = −4.050), and ASIh (−3.543).

Spearman’s Rho bivariate correlation analysis was performed to determine the relationships between the scores for the different questionnaires (see Table 5).

These results highlight the existence of significant relationships (*p* < 0.01) among the presence of sexual double standards, romantic love myths and sexist attitudes. The DSS was found to have a significant inverse correlation with excess scales, indicating that the lower the sexual double standards, the greater the romantic myths and sexism (benevolent and hostile). Likewise, if adherence to romantic myths increases, ambivalent sexist attitudes are reversed.

## 4. Discussion

The purpose of this study was to describe the presence of romantic love myths among adolescents, their expressions of sexist attitudes, and sexual double standards towards women. The specific objectives were: (1) to describe the differences in the above variables as a function of the sociodemographic characteristics of the sample and (2) to analyze the interrelationship between these variables. The three variables of ambivalent sexism, sexual double standards and romantic love myths were expected to be correlated with each other.

Regarding the first specific objective, the results obtained reflect significant differences (*p* < 0.01) according to gender, with boys (49.94%) presenting more sexist attitudes (both hostile and benevolent), greater adherence to the romantic love myths (in the idealization of love and in the union between love and abuse), and greater acceptance of sexual double standards. These results are consistent with those found in previous research, both in the context of the adolescent population and in the general population [31,32,33,34]. According to the study in adolescents by Navas et al. [31], ambivalent sexism is related to the Triple Dyad (Machiavellianism, psychopathy, and narcissism). 

In our study, adolescents who had a partner (64.16%) presented greater adherence to double sexual standards, romantic love myths and the subscale of the idealization of love, with significant differences in the EMA (*p* < 0.05) and EMAi (*p* < 0.01) scales. These results could be explained by the fact that when one person is in a couple, especially in adolescence, romantic love is idealized and the differences in sexual moral freedoms are intensified according to gender. There are other studies, such as those by Lara et al. [35] and Cava et al. [36], that study romantic love myths and the strength of the couple, or the correlation with dating violence or cyber dating violence, but these were carried out in adolescents with a partner, and the influence of being in or not being in a relationship could not be analyzed.

Regarding school grades, younger students and those in the first years of ESO (male and female = 53) were significantly more likely to present romantic love myths, sexist attitudes and sexual double standards (*p* < 0.01). When analyzing the effect size of the correlations, it can be observed that they were between medium and large, with regard to those occurring between the youngest compared to the oldest. These results may reflect the fact that sex education programs are taught in the last years of ESO. The programs are carried out by health personnel such as nurses and doctors, mainly through lectures and educational interventions [37]. These patterns of gender differentiation obtained in our results are consistent with those obtained by Solbes et al. [38] in children (4–9 years), where it was observed that, at this age, they have already internalized these discriminating aspects.

Regarding the correlations between sexual double standards, romantic love myths, and ambivalent sexism, the stronger the presence of sexual double standards, the weaker the presence of romantic love myths and sexist attitudes (Spearman’s Rho for DSS (*p* < 0.01)—EMA: −0.242; ASI: −0.493; ASIb: −0.431; ASIh: −0.446). This could indicate that gender-differentiated sexual freedom measures very different constructs from sexism and romantic love myths, although at first it seems that they could be concordant. On this basis, they should be studied in future research as separate and inverse constructs. On the other hand, the presence of romantic love myths was directly associated with sexist attitudes, both benevolent and hostile (Spearman’s Rho for EMA (*p* < 0.01)—ASI: 0.415; ASIb: 0.432; ASIh: 0.296). The correlation between romantic love myths (romantic jealousy) and sexist attitudes is in agreement with the study of Rodriguez-Domínguez et al. [33]; hostile sexism is related to dating violence, except that their study only concerned adolescent boys. A reduction in adolescent discourse of romantic love myths and sexist attitudes may be influenced by the socialization of relational processes. In other words, older adolescent boys and girls tend to be more rational, to distinguish more clearly between right and wrong. In addition, they feel more peer pressure at an older age. These factors provoke a change in attitudes, but do not have much influence on the construction of relationships, which are strongly impacted by socialization. In line with studies on gender-based violence and cyber violence in the general population, we found that in relationships, the presence of factors such as romantic love myths, sexist attitudes and sexual double standards facilitates gender-based violence, especially against women [31,36,39,40]. In summary, our findings show that factors measuring gender differentiation (sexism, sexual double standards, and romantic love myths) persist among boys and girls, even though society seems to be changing in this regard. Therefore, it is not enough to seek to change misconceptions; we must also address the fact that violence is a social problem, transmitted from generation to generation as part of the DNA of cultural socialization. Consequently, considerations of equality and mutual respect should be instilled from an early age by the school medical service in order to prevent and counteract the sexist violence that is still prevalent in our society [41,42].

The strengths of this study include the fact that it was conducted under optimal conditions of relaxation and concentration for the participants, without the imposition of authority. To our knowledge, this study is one of the few that examines a wide range of factors that, individually or together, may influence gender-based violence among such a young population, taking into account their relationship history. The study also has certain limitations. First, it is cross-sectional in nature and may be subject to selection bias resulting from the non-probability sampling conducted. In addition, the sample population was local. The study could be extended to secondary education and vocational institutions to determine whether extended higher education influences the study variables considered. In future research, moreover, we believe that longitudinal studies of these issues should be conducted and that educational interventions focused on these factors should be designed, tested, and implemented by specialists in the field of sexual health.

Among the implications of this study, we would like to highlight the importance of knowing that gender differentiating factors are still present and how they are interrelated. It is important to take them into account, especially in order to promote the healthy psychosocial development of young people, both individually and as couples, since distorted ideas influence their development [31]. Likewise, this study offer clues for health personnel, who provide educational interventions, and can give guidance at the political level, as regards the construction of educational laws and which aspects we must continue to educate about to eradicate these concepts. This would perhaps indirectly reduce gender violence against women from an early age.

## 5. Conclusions

The adolescents in our study population presented romantic love myths, sexist attitudes and sexual double standards, a pattern that was especially evident among the male population. There were significant correlations among these three factors, all of which may influence the appearance of gender violence. To address this problem, it is important for adolescents to receive appropriate sexual education, addressing among other questions the parameters that may induce violence against women in intimate relationships.

## Figures and Tables

**Table 1:** Descriptive analysis of qualitative variables.

		*n*	%
Gender	Male	439	49.94%
	Female	440	50.06%
School year	1st ESO	53	6.03%
	2nd ESO	136	15.47%
	3rd ESO	347	39.48%
	4th ESO	343	39.02%
Partner	Yes	564	64.16%
	No	312	35.50%
	No reported	3	0.34%
Sexual orientation	Heterosexual	821	95.69%
	Homosexual	16	1.86%
	Bisexual	21	2.45%
Location	Urban	647	73.61%
	Rural	232	26.39%

Note—%: percentages; ESO: compulsory secondary education.

**Table 2:** Descriptive analysis of quantitative variables.

	Mean (SD)	CI 95%
Age	14.85 (1.32)	(14.79, 14.95)
Age of first relationship	14.53 (1.19)	(14.35, 14.70)
DSS	34.01 (7.20)	(33.53, 34.50)
EMA	24.50 (6.50)	(24.46, 25.33)
EMAi	20.86 (4.76)	(20.54, 21.18)
EMAa	4.03 (2.89)	(3.84, 4.22)
ASI	54.74 (18.87)	(53.45, 56.03)
ASIb	27.45 (10.61)	(26.74, 28.17)
ASIh	27.41 (10.60)	(26.70, 28.13)

Note—M: mean scores; SD: standard deviation; CI 95%: confidence interval 95%; DSS: Double Standard Scale; EMA: Romantic Love Myths Scale; EMAa: link between love and abuse; EMAi: idealization of love; ASI: Ambivalent Sexism Inventory; ASIb: Benevolent Sexism Scale; ASIh: Hostile Sexism Scale.

**Table 3:** Mean scores and standard deviations for each scale, according to academic year, gender, relationship status and location.

		DSS		EMA		EMAi		EMAa		ASI		ASIb		ASIh	
		M	SD	M	SD	M	SD	M	SD	M	SD	M	SD	M	SD
School year	1st ESO	29.82	4.28	29.75	9.04	22.21	6.91	7.54	2.93	68.93	8.19	34.97	5.34	33.76	5.46
	2nd ESO	32.61	7.11	26.64	6.61	22.05	4.89	4.60	3.04	59.71	17.62	29.64	10.36	30.02	9.09
	3rd ESO	34.60	7.10	24.56	5.46	20.90	4.11	3.66	2.65	55.93	18.73	28.03	10.54	26.89	10.65
	4th ESO	34.58	7.46	23.78	6.52	20.15	4.80	3.63	2.66	50.72	19.17	24.97	10.60	25.98	11.19
Gender	Male	32.88	6.51	25.37	6.85	20.93	4.83	4.43	3.11	59.46	16.00	28.73	9.70	30.80	8.97
	Female	35.09	7.68	24.41	6.12	20.77	4.70	3.63	2.59	50.00	20.33	23.95	11.03	26.25	11.30
Partner	Yes	34.22	7.33	25.20	6.39	21.23	4.67	3.97	2.84	54.64	18.36	27.47	10.39	27.38	10.45
	No	33.67	7.02	24.25	6.64	20.14	4.86	4.11	2.96	54.68	19.88	27.40	11.08	27.27	10.81
Location	Urban	33.32	7.31	25.82	6.94	21.30	5.01	4.51	3.11	56.46	17.83	28.34	10.13	28.23	9.88
	Rural	35.91	6.54	22.35	4.17	19.65	3.73	2.70	1.55	50.10	20.78	25.04	11.52	25.14	12.11

Note—DSS: Double Standard Scale; EMA: Romantic Love Myths Scale; EMAi: idealization of love; EMAa: link between love and abuse; ASI: Ambivalent Sexism Inventory; ASIb: Benevolent Sexism Scale; ASIh: Hostile Sexism Scale; M: mean; SD: standard deviation; ESO: compulsory secondary education.

**Table 4:** Kruskal–Wallis test adjusted with the Bonferroni correction for school year and Cohen’s d.

School Year	DSS		EMA		EMAi		EMAa		ASI		ASIb		ASIh	
	H	ES	H	ES	H	ES	H	ES	H	ES	H	ES	H	ES
1st–2nd	−112.03 **	−0.43	84.09		3.15		219.56 *	0.98	136.72 *	0.59	137.67 *	0.57	102.73	0.82
1st–3rd	−183.36 *	−0.70	161.60 *	0.86	63.21		297.45 *	1.44	204.46 *	0.78	179.55 *	0.69	178.18 *	0.68
1st–4th	−181.74 *	−0.67	206.42 *	0.86	108.43 **	0.40	303.84 *	1.45	255.66 *	1.00	250.73 *	0.99	192.20 *	0.73
2nd–3rd	−71.32 **	−0.28	77.50 **	0.36	60.06		23.22*	0.34	67.73 **	0.26	41.88	0.15	75.45 **	0.31
2nd–4th	−69.71 **	−0.27	122.33 *	0.44	105.28 *	0.16	23.26 *	0.35	118.94 *	0.48	113.05 *	0.44	89.46 *	0.38
3rd–4th	1.61		44.826		45.23		17.42		51.20 **	0.22	71.17*	0.29	14.01	

Note—H: statistical Kruskal–Wallis; ES: effect size (Cohen’s d); DSS: Double Standards Scale; EMA: Romantic Love Myths Scale; EMAa: link between love and abuse; EMAi: idealization of love; ASI: Ambivalent Sexism Inventory; ASIb: Benevolent Sexism Scale; ASIh: Hostile Sexism Scale; Z: statistic; * *p* < 0.01; ** *p* < 0.05.

**Table 5:** Spearman’s Rho coefficient of correlation.

		EMA	EMAi	EMAa	ASI	ASIb	ASIh
DSS	Coefficient of correlation	−0.372 *	−0.422 *	−0.262 *	−0.463 *	−0.396 *	−0.419 *
EMA	Coefficient of correlation		0.630 *	0.915 *	0.395 *	0.354 *	0.327
EMAi	Coefficient of correlation			0.315 *	0.352 *	0.329 *	0.273 *
EMAa	Coefficient of correlation				0.267 *	0.204 *	0.273 *
ASI	Coefficient of correlation					0.872 *	0.859 *
ASIb	Coefficient of correlation						0.531 *

DSS: Double Standard Scale; EMA: Romantic Love Myths Scale; EMAi: idealization of love; EMAa: link between love and abuse; ASI: Ambivalent Sexism Inventory; ASI: Ambivalent Sexism Inventory; ASIb: Benevolent Sexism Scale; ASIh: Hostile Sexism Scale. * *p* < 0.01.

## Data Availability

The data were generated during the study.
